# The Role of Co-stimulatory/Co-inhibitory Signals in Graft-vs.-Host Disease

**DOI:** 10.3389/fimmu.2018.03003

**Published:** 2018-12-21

**Authors:** Sandeep Kumar, Nicholas D. Leigh, Xuefang Cao

**Affiliations:** ^1^Department of Immunology, Roswell Park Comprehensive Cancer Center, Buffalo, NY, United States; ^2^Department of Microbiology and Immunology, Marlene and Stewart Greenebaum Comprehensive Cancer Center, University of Maryland, Baltimore, MD, United States

**Keywords:** allogeneic hematopoietic cell transplantation (allo-HCT), graft-vs.-host disease (GVHD), graft -vs.-leukemia (GVL) effect, T cells, co-stimulation/co-inhibition

## Abstract

Allogeneic hematopoietic cell transplantation (allo-HCT) is an effective immunotherapeutic approach for various hematologic and immunologic ailments. Despite the beneficial impact of allo-HCT, its adverse effects cause severe health concerns. After transplantation, recognition of host cells as foreign entities by donor T cells induces graft-vs.-host disease (GVHD). Activation, proliferation and trafficking of donor T cells to target organs and tissues are critical steps in the pathogenesis of GVHD. T cell activation is a synergistic process of T cell receptor (TCR) recognition of major histocompatibility complex (MHC)-anchored antigen and co-stimulatory/co-inhibitory signaling in the presence of cytokines. Most of the currently used therapeutic regimens for GVHD are based on inhibiting the allogeneic T cell response or T-cell depletion (TCD). However, the immunosuppressive drugs and TCD hamper the therapeutic potential of allo-HCT, resulting in attenuated graft-vs.-leukemia (GVL) effect as well as increased vulnerability to infection. In view of the drawback of overbroad immunosuppression, co-stimulatory, and co-inhibitory molecules are plausible targets for selective modulation of T cell activation and function that can improve the effectiveness of allo-HCT. Therefore, this review collates existing knowledge of T cell co-stimulation and co-inhibition with current research that may have the potential to provide novel approaches to cure GVHD without sacrificing the beneficial effects of allo-HCT.

## Introduction

Hematopoietic cell transplantation (HCT) is a proven therapeutic approach for patients suffering from various hematologic and immunologic diseases (1). HCT is an immunotherapy procedure during which a healthy donor provides hematopoietic cells including stem and progenitor cells to a diseased host. Transplantation of donor-derived cells is an attempt to re-establish hematopoietic and immunological activities in the host. HCT is effective for a number of diseases including various hematologic malignancies, non-cancerous diseases including aplastic anemia, thalassemia, sickle cell anemia, and severe combined immunodeficiency (2). Apart from hematologic malignancies such as leukemia, lymphoma, and myeloma, HCT showed positive yet limited effects in patients taking chemotherapeutic regimens for solid tumors. For example, uses of HCT with anticancer regimens were reported to be effective in germ cell tumors, soft tissue sarcomas, and neuroblastoma (3).

Based on the use of conditioning regimens, HCT can be categorized into myeloablative and non-myeloablative transplantation. In myeloablative transplantation, patients usually go through a high dose of chemotherapy or radiation exposure prior to HCT while non-myeloablative transplantation is a reduced intensity transplantation procedure that is performed after less intensive chemotherapy (4). Based on human leukocyte antigen (HLA) phenotyping, HCT can be categorized into allogeneic HCT (allo-HCT) and syngeneic HCT (syn-HCT). Allo-HCT is a potentially curative procedure, for a variety of health concerns including cancerous and non-cancerous conditions (5). Based on HLA phenotyping and source of donor hematopoietic cells, allo-HCT can be further divided into match related, match unrelated and haploidentical HCT. In match related allo-HCT, the donor is a biological family member that bears 10 major HLAs identical to the host. In match unrelated allo-HCT, the donor is genetically unrelated yet possesses 10 major HLAs identical to the host. Haploidentical donor is typically a family member with HLAs half-matched to the host. All of these major types of allo-HCT have demonstrated beneficial effects in patients. However, a major limitation is the prevalence of adverse effects in the host known as graft- vs.-host disease (GVHD), which remains a major cause of morbidity and mortality in allo-HCT patients (6). In allo-HCT, donor immune cells recognize the host as a foreign entity and subsequently attack and damage normal host tissues and organs. Based on the occurrence timeline and target organs, GVHD is divided into acute GVHD (aGVHD) and chronic GVHD (cGVHD). Prevalence of aGVHD is associated with factors including differences in HLA phenotypes (7), previous pregnancy of donor and advanced age of either the donor or the recipient (8). Prevalence of cGVHD can occur due to HLA-mismatched donor or from an HLA-matched unrelated donor (9), or patients that may have already experienced aGVHD (10). Patients with aGVHD who fail to respond adequately to corticosteroids are known as steroid resistant (SR) and require salvage treatment, with anti-T cell antibodies being the most commonly utilized group of agents (11). These currently practiced clinical strategies have shown limited success in controlling GVHD. Therefore, novel approaches including those targeting T cell activation are being vigorously pursued in order to cure GVHD.

Donor-derived T cells play a central role in GVHD (12). The inflammatory cytokines produced by allo-reactive effector CD4^+^ and CD8^+^ T cells are involved in the pathogenesis of GVHD (13). Cytokines produced by the conditioned host cells and donor T cells contribute to allo-reactive T cell activation, proliferation and trafficking to the target organs including skin, gut and liver (14). Aside from soluble cytokines, direct contact-dependent cytotoxic damage of host cells by donor T cells also contributes to the pathology of GVHD (15, 16). Once activated, allo-reactive effector T cells migrate to target tissues, where they deliver their destructive potential mediated by Fas ligand, TNF-α, TNF-related apoptosis-inducing ligand (TRAIL), perforin, granzymes (Gzm), and IFN-γ that lead to apoptosis in epithelial target cells (17). Later, allo-reactive memory T cells are generated and cause persistent host tissue injury (18). The damage inflicted by donor T cells provides the rationale for T cell depletion (TCD), which has been shown to be effective in the prevention of GVHD (19). However, overall TCD eliminates all T cell subsets and leads to defective immunity that results in a disease prone host. Therefore, there is an urgent need for a therapeutic approach that attenuates allo-reactive T cell function without compromising overall T cell immunity.

T cell activation is a complex yet highly regulated process. Binding of T cell receptor (TCR) to the major histocompatibility complex (MHC)-anchored antigen peptides is the first step of T cell activation. However, this activation is not optimal in the absence of co-stimulatory signals. Co-stimulatory molecules potentially regulate various functions of T cells including activation, proliferation, differentiation and survival. Common examples of co-stimulatory molecules are CD28, ICOS, CD40, CD30, CD27, OX40, and 4-1BB. Optimal activation of T cells comprises various inter-cellular and intra-cellular events including engagement of TCR, recruitment of tyrosine kinases to TCR complex, subsequent signal transduction into the nucleus and initiation of transcription and translation. On the other hand, negative regulation of T cell activation is mediated by co-inhibitory signals such as CTLA-4, PD-1, TIM-3, and LAG-3 (20). During GVHD development, co-stimulatory/co-inhibitory molecules are involved in the functional alloreactivity of immune cells which is associated with up-regulated expressions and activities of several co-stimulatory/co-inhibitory signals (21). For example, expressions of co-stimulatory molecules CD134 (OX40) and CD154 (CD40 Ligand) are up-regulated on CD4^+^ and CD8^+^ T cells in aGVHD patients (21). In this review, we explore various components of co-stimulatory/co-inhibitory pathways in the setting of allo-HCT and aim to illuminate their potential roles in GVHD. This review also attempts to discuss co-stimulatory/co-inhibitory molecules that can be targeted as potential therapeutic options for GVHD.

## Relevance of T Cell Co-stimulation/Co-inhibition Molecules in GVHD

A cascade of cellular and molecular interactions are responsible for T cell activation, differentiation, migration, and effector function during GVHD. In addition to TCR-mediated “signal one,” co-stimulatory molecules provide “signal two” that is essential to fully activate T cells while avoiding anergy (22). In the form of “signal two,” the involvement of various molecular pathways can lead to positive as well as negative regulation of T cell function. Therefore, they have been classified as *co-stimulatory* and *co-inhibitory* signals. The majority of co-stimulatory/co-inhibitory molecules belong to either immunoglobulin superfamily (Ig-SF) or TNF receptor superfamily (TNFR-SF). Both of these receptor families are integral in T cell regulation and are dynamically and temporally regulated. In addition, there are several other co-stimulatory molecules that are different in structure and functions when compared to Ig-SF and TNFR-SF. One example is the nectin and nectin-like co-stimulatory family. Here we summarize the roles of various co-stimulatory/co-inhibitory molecules in the pathogenesis of GVHD.

### Ig-SF Co-signaling Molecules

Many Ig-SF members have been thoroughly studied for their involvement in the activation, tolerance, and functionality of T cells. The best known Ig-SF members include CD28, cytotoxic T-lymphocyte-associated protein 4 (CTLA-4), B7-1 (CD80), B7-2 (CD86), inducible co-stimulator (ICOS), B7-H2, and programmed cell death protein 1 (PD-1), B7-H1 (PD-L1), and lymphocyte-activation gene 3 (LAG-3) (23). Here, we will discuss their roles in the context of GVHD.

Because CD28-mediated co-stimulation has an important role in the initiation and maintenance of T cell response, several studies were carried out to explore whether CD28 is critical for the development of GVHD. These studies demonstrated that CD28 is involved in GVHD and the severity of GVHD could be decreased by the administration of agents that block CD28 function (24, 25). Beneficial outcomes in GVHD due to the interruption of CD80/CD28 axis are well-established (24). Using anti-B7-1 (also known as CD80) plus anti-B7-2 (also known as CD86) monoclonal antibodies, it was demonstrated that B7-1 expression on donor T cells is critical for maximal GVHD lethality induced by either CD8^+^ or CD4^+^ T cells (24). This outcome was later corroborated by another approach advocating antisense gene therapy targeting B7-1 that resulted in diminished rejection of allogeneic graft (26). Another notable finding is that a CD28 superagonist has the ability to decrease GVHD via increasing immunosuppressive T regulatory (Treg) cells (27). This further emphasizes the complexity of modulating co-stimulation in GVHD. However, this finding will unlikely be clinically applicable due to the catastrophic clinical trial with CD28 superagonist (28, 29).

ICOS (CD278) is a member of Ig-SF expressed on activated T cells that contributes to the induction of GVHD in the absence of B7/CD28 co-stimulation (30). Blocking of CD28 and ICOS while sparing CTLA-4 represents a promising approach to abrogate pathogenic T cell response following allo-HCT (30). It was reported that interaction between B7-related protein-1 (B7RP-1) and ICOS is important because blockade of this interaction suppresses allo-reactive T cells and reduces lethal aGVHD (31). However, a surprising result was that ICOS played differential roles in CD4^+^ and CD8^+^ T cell-mediated GVHD (32). ICOS deficiency was found to increase CD8^+^ T cell mediated GVHD, while it played the expected role in CD4^+^ T cells—that is, decreased GVHD with ICOS deficiency. Intercellular adhesion molecule (ICAM) is also a member of Ig-SF that binds to lymphocyte function-associated antigen 1 (LFA1) receptor. Blocking of CD28/B7 and LFA1/ICAM pathways can effectively prevent GVHD in MHC-mismatched mouse models (33).

In contrast to these co-stimulatory Ig-SF members, there are several Ig-SF members that induce inhibitory effects on T cell activation and function. CTLA-4 possesses similar structure to CD28. Due to this structural similarity, CTLA-4 acts as a competitor to CD28 (34, 35). An intriguing study demonstrated that lethality of aGVHD is highly dependent on CD28/CTLA-4 competition (34). Use of CTLA4-Ig has been found to improve survival rate in mice suffering from GVHD (36). Since CD4^+^CD25^+^ Treg T cells constitutively express CTLA-4 and activated T cells express B7-1 and B7-2, interaction between CTLA-4 and B7-1/B7-2 among T cells may represent an important mechanism for suppression (37). Also, donor Treg cells interact with host APCs via B7-1/B7-H1 but not PD-1/B7-H1 axis that augments donor Treg survival and expansion (38). B7-H4 is another co-inhibitory molecule that has shown an important role in GVHD (39). B7-H4 expressed on human bone marrow-derived mesenchymal stem cells inhibits T cell activation and proliferation via induction of cell cycle arrest and inhibition of NF-κB nuclear translocation (39). PD-1 (CD279) is another co-signaling molecule that induces an inhibitory effect on T cell activation and proliferation. PD-1 has also demonstrated an important role in the suppression of GVHD (40). PD-1H is a subtype of PD-1 that was recently identified as an Ig-SF co-inhibitory molecule, with a study showing that a single injection of PD-1H agonistic MoAb protects mice from GVHD (40). LAG-3, also known as CD223, is a type I transmembrane protein with four extracellular Ig-like domains. Elimination of LAG-3 signaling resulted in increased GVHD (41).

In summary, most of these studies concluded that blocking Ig-SF co-stimulatory molecules had the ability to decrease GVHD, while abrogation of Ig-SF co-inhibitory signaling increases GVHD. However, complicated and differential roles were reported regarding the function of some receptor/ligand pairs on various T cell subsets. Further studies are required for exploitation of these molecules as therapeutic targets.

### TNFR-SF Co-signaling Molecules

TNFR-SF is another extensively studied co-signaling receptor super family involved in T cell activation. TNFR-SF members bind to TNF-SF ligands and mediate their action via downstream signaling molecules including TNFRSF1A-associated death domain (TRADD), TNF receptor associated factors (TRAF), TNF-dependent recruitment of the protein kinase (RIP) and Fas-associated protein with death domain (FADD) (42). TNFR-SF members include TNFR1, TNFR2, OX40, CD40, Fas, decoy receptor 1, 2, and 3, CD27, CD30, 4-1BB, death receptor 3 (DR3), DR4, DR5, and DR6, receptor activator of nuclear factor κB (RANK), osteoprotegerin (OPG), TNF-like weak inducer of apoptosis (TWEAK) receptor, trans membrane activator and CAML interactor (TACI), herpes virus entry mediator (HVEM), glucocorticoid-induced TNF receptor (GITR), orphan receptor in the TNF family (TAJ), and receptor expressed in lymphoid tissues (RELT). Here, we will discuss how various TNFR-SF members are involved in GVHD.

OX40 (also known as CD134) was reported as an option to attenuate GVHD aggravation because it has negative baseline expression yet rapid upregulation after activation (43). OX40 is an activation-induced co-stimulatory molecule, expressed by activated CD4^+^, CD8^+^ T cells and Treg cells after TCR ligation (44, 45). OX40L (also known as CD134L) is the binding target for OX40. APCs including DCs, B cells, and macrophages express OX40L on their surface. CD40 or LPS stimuli are important for the activation of OX40/OX40L axis (44, 45). Several studies suggest a potential role of OX40/OX40L axis in GVHD using various approaches such as antagonistic anti-OX40L MoAb or OX40KO donor or OX40LKO host mice (44–46). The depletion of OX40 from allogeneic graft has been found to suppress GVHD severity without hampering GVL effect or immunity against infectious pathogens (43). Although both CD4^+^ and CD8^+^ T cells demonstrate elevated expression of OX40, major OX40 effect was observed in CD4^+^ T cell-mediated GVHD. Of note, the OX40/OX40L axis is not directly linked to CD28/B7 pathway (46, 47). Interestingly, triggering the OX40/OX40L axis on CD4^+^CD25^+^ Treg cells may block their suppressive function (45). A similar study suggests that OX40 blockade might be crucial to optimize the use of Treg cells to prevent GVHD (48).

CD137 (TNFRSF9), commonly known as 4-1BB, is an inducible type I membrane protein of TNFR-SF (49). 4-1BB plays an important role in co-stimulation of CD4^+^ and CD8^+^ T cells following antigenic or mitogenic activation (50). CD8^+^ T cells are more responsive to early activation and proliferative signals trigged by the TCR and 4-1BB, while the function of the 4-1BB/4-1BBL axis is reciprocal to the CD28/B7 co-stimulatory signals (49). Several studies suggest that 4-1BB plays an important role in GVHD (49, 51, 52). In a murine model of aGVHD, it was observed that administration of epitope-specific anti-4-1BB MoAb increases host-reactive cytotoxic T cell population (49). In addition, *in vitro* exposure of donor T cells to 4-1BBL MoAb may attenuate GVHD (51).

CD40 is a member of TNFR-SF expressed mainly on APCs that binds to CD154 (also known as CD40L) expressed on T cells (53). Blockade of CD40/CD40L was found to decrease T cell-mediated GVHD (54, 55). CD40L deficiency can be exploited for GVHD management (56). In a murine model of cGVHD, an agonistic CD40 moAb prevented donor CD8^+^ T cell anergy. Subsequently activated donor CD8^+^ T cells deleted host CD4^+^ T cells and host B cells involved in autoantibody production, leading to decreased cGVHD (57). Furthermore, activated donor CD8^+^ T cells induced full engraftment of donor hematopoietic cells and exhibited an increased GVL effect (57). In addition, simultaneous use of CD40 and CD28 antagonists has shown a benefit in the attenuation of aGVHD (58). Both CD40-activated B cells and immature DCs can function as professional APCs to induce antigen-specific Treg cells (59). However, CD40-activated B cells are more potent in expanding Treg cells which is more efficient in attenuating GVHD (59). Together, these studies suggest a therapeutic potential for targeting CD40 in GVHD management.

CD27 receptor is an important member of TNFR-SF that is required for the generation and long-term maintenance of T cell immunity (60). It binds to ligand CD70, and plays a key role in regulating B cell activation and immunoglobulin synthesis (60). A study carried out with CD27 knockout mice reveals that CD27 is essential in CD4^+^ and CD8^+^ T cell activation and memory formation (61). A clinical study showed that patients who developed cGVHD had proportional increase in CD27^+^ B cells in the first year after HSCT (62). However, our recent studies with mouse models demonstrate that CD27/CD70 pathway surprisingly provides immunosuppressive signaling during GVHD. The absence of CD27/CD70 signaling in both donor T cells and the host significantly increases T cell expansion and effector function, which subsequently leads to increased GVHD lethality (63, 64). Another surprising and complex result was found with GITR, with GITR^−/−^ CD4^+^ T cells mediating increased GVHD vs. WT controls and GITR^−/−^ CD8^+^ T cells showing decreased alloreactivity (65).

Taken together, members of TNFR-SF have demonstrated significant therapeutic potential in GVHD. However, results also show that these co-signaling molecules form a complex system. Further methodical and extensive study is needed to fully delineate the roles for these co-stimulatory and co-inhibitory molecules in GVHD.

### Other Co-signaling Molecules

Apart from the two major co-stimulatory/co-inhibitory super families, there are several other co-stimulatory molecules that are structurally or functionally different. Nectin and nectin-like (Necl) molecules are immunoglobulin like type I trans membrane glycoproteins that possess property of Ca++ independent cellular adhesion (66). The known members of this family are Nectin-1, Nectin-2, Nectin-3 and Nectin-4, Nectin-5, Necl-1, Necl-2, Necl-3, Necl-4, and Necl-5.

Thus far, literature is not abundant for the role of Nectins or Necls in GVHD. Nectin-2 is a ligand for DNAX accessory molecule (DNAM-1, also known as CD226) and involved in NK and T cell-mediated cytotoxicity (67). DNAM-1 is involved in regulating NK cell IFN-γ production and cytotoxicity against various cancer and infected cells (68). NK cells suppress GVHD by attenuating activation of alloreactive T cells without hampering GVL effect (69). Thus, manipulating Nectins and Necls on NK and T cells may represent a novel approach to manage GVHD. In support of this rationale, absence of DNAM-1 on the donor graft attenuates GVHD in MHC-mismatched and MHC-matched allo-HCT, whereas it is not critical for GVL effect against CD155 (another DNAM-1 ligand)-expressing and CD155 non-expressing leukemias. In addition, absence of DNAM-1 promotes the expansion and suppressive function of Treg cells after allo-HCT (70).

Cytotoxic and regulatory T cell molecule (CRTAM) is a member of Necls family. It is a MHC I-restricted T cell associated molecule and its expression is restricted to activated NKT and CD8^+^ T cells (71, 72). Interestingly, CD4^+^ T cells that express CRTAM upon activation gained the characteristics of CD8^+^ T cells. Further analysis of CRTAM^+^CD4^+^ T cells revealed IFN-γ production, expressions of CTL-related genes like eomesodermin, GzmB, and perforin, and cytotoxic function. Furthermore, CRTAM^+^ T cells traffic to mucosal tissues and inflammatory sites where they release IFN-γ and deliver cytotoxic activity (73). These features would make it interesting to study the possible involvement of CRTAM in GVHD.

Leukocyte immunoglobulin (Ig)-like receptors (LILRs) or immunoglobulin-like transcript (ILT) family or CD85 genes are a family of inhibitory and stimulatory receptors (74). Several members from this family have been discovered including ILT2, ILT3, and ILT5. ILT2 is expressed on activated T cells and may function to shut down T cell activation, culminating in T cell death or induction of anergy (75). The expression of HLA-G (an ILT2 ligand) during allogeneic recognition is associated with better graft acceptance (76). The interaction of HLA-G with ILT2 is associated with immunosuppressive mechanisms that require expansion of myeloid-derived suppressor cells (MDSCs). Induction of MDSCs by ILT2/HLA-G axis can prevent allograft rejection (77). Another member of ILT family, ILT3, is crucial for the tolerogenic activity acquired by DCs exposed to allogeneic antigen-specific CD8^+^ T suppressor cells (78). A derivative of ILT3, ILT3-Fc, can serve as a potent immune regulatory agent that attenuates allograft rejection in humanized NOD/SCID mice by induction of CD8^+^ T suppressor cells (79). A recent study suggests that a subset of allo-HCT recipients generate antibodies directed to surface molecules of DCs, in particular ILT5. The ILT5-specific antibodies can mediate depletion of ILT5-bearing cells. ILT5 expression has been observed in some leukemic cells, indicating that it might be a target for GVL effect (80).

T cell immunoglobulin mucin (Tim) family members regulate immune responses, autoimmunity, and allergy (81). Members of Tim family have also been reported to be involved in GVHD (82, 83). Tim-3 is a member of Tim family expressed on Th1 cells. Tim-3 binds to galectin-9 and negatively regulates Th1 response. During immune homeostasis, Tim-3 interaction with galectin-9 leads to the deletion of Tim-3^+^ T cells. Tim-3 is up-regulated on activated T cells during GVHD (84). Blockade of Tim-3/galectin-9 interaction by infusion of a Tim-3-Ig fusion protein or Tim-3 knockout in donor T cells increases T cell proliferation and GVHD lethality (84). Inhibition of Tim-3 in aGVHD augments the activation of effector T cells expressing IFN-γ or exerting cytotoxic activity (82). A proteomic study also identifies increased levels of soluble Tim-3 in plasma of subjects with mid-gut and upper-gut GVHD (83).

Taken together, other co-signaling molecules besides Ig-SF and TNFR-SR may be important regulators of T cell function during allo-HCT. These molecules add complexities to T cell co-signaling that we need to comprehensively study to explore their therapeutic potential in GVHD.

## Co-Signaling Receptor Signal Transduction Pathways in GVHD

T cell activation is triggered by two signals (TCR/MHC and co-stimulatory) in the presence of cytokines. Activation of TCR and subsequent engagement of CD4 or CD8 co-receptor induce the recruitment of tyrosine phosphatase CD45 (85), which dephosphorylates Src family tyrosine kinases FYN and lymphocyte protein tyrosine kinase (LCK) (86). Activation of LCK resulted in phosphorylation of immunoreceptor tyrosine-based activation motif (ITAM) on CD3 in the TCR complex (86). Phosphorylation of ITAMs activates zeta-chain associated protein kinase-70 (ZAP-70). Activation of ZAP-70 leads to phosphorylation of ZAP-70 substrates including adapters like SLP76 and inducible T cell kinase (ITK). ITK phosphorylates phospholipase C γ1 (PLCγ1) that leads to the hydrolysis of phosphatidylinositol 4, 5-bisphosphate (PIP2) and second messengers diacylglycerol (DAG) and inositol trisphosphate (IP3). DAG activation leads to subsequent activation of PKC-θ that induces MAPK/ERK pathways and ultimately leads to the activation of transcription factor NF-κB (86). IP3 causes release of Ca++ from the endoplasmic reticulum that promotes influx of external Ca++ into the cells due to the formation of Ca++ dependent channel (87, 88). Then Ca++ binds to calmodulin and activates calcineurin (a phosphatase) that up-regulates transcription of IL-2 through NFAT (87, 88). These signaling events set in motion various immune responses including antibody production, activation of phagocytic cells and direct cell killing (87, 88). In this section we will describe how various co-stimulatory/co-inhibitory signal transduction pathways contribute to GVHD.

The CD28/B7 pathway is highly important in the pathogenesis of GVHD. Several studies were performed to understand the role of CD28/B7 axis in signal transduction (89–91). The patterns of tyrosine phosphorylation in T cells triggered by CD28 interaction with B7-1 and B7-2 are identical, but different from the tyrosine phosphorylation induced by TCR-MHC interaction (89). The major difference is adapter protein Grb2 that is regulated by TCR both *in vivo* and *in vitro* whereas *in vivo* study reveals no apparent regulation of Grb2 complex in response to B7-1 or B7-2 (89). The other unique protein is adaptor protein p62 that is phosphorylated in B7-1 and B7-2 signaling but not in TCR signaling (89). B7-1 and B7-2 do not activate Raf-1/ERK2 cascade in MAP kinase pathway. Instead, B7-1 and B7-2 cooperate with intracellular Ca++ increase and PKC activation to stimulate Jun kinases (90). CD28 binding to B7 contributes to setting the level of TCR-induced phosphorylated LAT for recruiting signaling complexes, while CD28 signaling further boosts multiple pathways by facilitating PLCγ1 activation (91). A recent work revealed that CD28 and ITK signaling regulate the trafficking of self-reactive T cells to target tissues in an autoimmune disease model, and pharmacological inhibition of ITK prevented this trafficking (92). Another co-stimulatory molecule, ICOS potentially activates PI3K pathway (93). ICOS signal transduction has been studied in GVHD. It was observed that PI3K-independent ICOS signaling mechanisms contribute to T cell co-stimulation during GVHD. Interestingly, CD4^+^ T cells preferably transduce via ICOS/PI3K signaling pathway, whereas CD8^+^ T cells induce GVHD in a PI3K-independent ICOS signaling mechanism (93). The OX40/OX40L axis induces activation of PLC signal transduction pathway (94). Interestingly, *in vivo* blockade of OX40/OX40L axis inhibited GVHD via a mechanism that did not require CD28 signaling, Stat-4, or Stat-6 signaling (47). CD27 is another member of TNFR-SF co-stimulatory family that is involved in GVHD. The CD27/CD70 axis transduces signals leading to the activation of NF-κB and MAPK8/JNK signaling via TRAF2 and TRAF5 (61). However, in the setting GVHD, literature is still inadequate on how these signal transduction pathways regulate allogeneic T cell response.

On the other hand, co-inhibitory molecules are highly important in immune regulation during GVHD. CTLA-4 ligation has been reported to downregulate activity of transcription factors including AP-1, NFAT, and NF-kB in activated CD4^+^ T cells. Reduced DNA-binding by AP-1 and NFAT complexes in the nucleus was observed due to CTLA-4 ligation (95). PD-1 signaling attenuates various steps of T cell signaling by TCR including phosphorylation of ZAP70 and PKCθ activation (96). CTL-4 ligation induces inhibitory effect on AKT but not on PI3K activation (97). In contrast, PD-1 signaling inhibits PI3K activity (96). These results support the current model of T cell co-stimulation vs. co-inhibition in which CD28 signaling promotes GVHD whereas CTLA-4 signaling inhibits GVHD (98). Interestingly of note, it appears that the inducible co-stimulatory ICOS and the co-inhibitory PD-1 may converge on PI3K to modulate T cell response.

Altogether, these co-signaling receptor signal transduction pathways may play a very important role in GVHD but further studies are required to exploit these pathways for more effective therapeutic intervention.

## Therapeutic Regimens in GVHD Management

Currently, a spectrum of therapeutic regimens are available to treat GVHD. Several drugs are used before and after allo-HCT to suppress allogeneic immune response. For examples, glucocorticoids including methylprednisolone and prednisone are commonly used and can effectively control GVHD in some patients. Mycophenolate mofetil, cyclosporine, and methotrexate (MTX) are also used to manage GVHD (99). Methylprednisolone or prednisone has also been used in combinations with other drugs including cyclosporine and MTX to control aGVHD (100). Other regiments including antithymocyte globulin, denileukin diftitox, infliximab, sirolimus, and tacrolimus are available now or are in clinical trials as supplemental drugs to standard treatment. However, steroid resistance (SR) in GVHD patients has been a rising concern. Therefore, studies are underway to investigate therapeutic options that can ameliorate GVHD in SR patients. Several examples of such drugs are daclizumab, etanercept, extracorporeal photopheresis, infliximab, pentostatin, rituximab, tacrolimus, thalidomide, and imatinib mesylate. Although these drugs are effective to control GVHD to a certain extent, detrimental side effects are still common and serious. These side effects include diarrhea, nausea, infection, diabetes, psychosis, insomnia, anemia, renal dysfunctions, neurotoxicity, hypertension, infusion reactions, hepatitis reactivation, hypertriglyceridemia, renal insufficiency, and cytopenia (101). Considering these severe side effects caused by the current therapeutic regimens, there is an urgent need for novel therapeutic interventions with minimal toxicity in GVHD. In response to this need, clinical trials are being carried out targeting co-signaling molecules to prevent or treat GVHD. Several ongoing trails are listed as a summary in Table 1.

**Table 1:** Currently ongoing clinical trials involving co-stimulatory or co-inhibitory signals.

**Status**	**Study title**	**Co-signaling molecules involved**
Active, not recruiting	Adoptive immunotherapy with activated marrow infiltrating lymphocytes and cyclophosphamide graft-vs.-host disease prophylaxis in patients with relapse of hematologic malignancies after allogeneic hematopoietic cell transplantation	Using anti-CD3/CD28 activated lymphocytes as treatment of relapse after allo-HCT for patients with hematologic malignancies
Recruiting	Abatacept for GVHD prophylaxis after hematopoietic stem cell transplantation for pediatric sickle cell disease	Co-inhibitory abatacept (CTLA4-Ig) added to standard GVHD prophylaxis regimen
Not yet recruiting	CD40-L blockade for prevention of acute graft-vs.-host disease	CD40-L blockade for prevention of GVHD
Active, not recruiting	Bridging Pediatric and Adult Biomarkers in graft-vs.-host disease	ST2 as a predictive biomarker for GVHD diagnosis and prognosis

## Recent Advancements in ALLO-HCT

Allo-HCT studies have been very productive in recent years, with discovery of new drug targets and diagnostic approaches. One example is aurora kinase A (AURKA). This kinase is associated with cellular division and proliferation and its defective form is associated with cancer. A recent study carried out a comprehensive elucidation of T cell transcriptome in non-human primate aGVHD. Results suggest that AURKA can be a potential target for preventing GVHD (102). Another newly introduced therapeutic target is soluble suppression of tumorigenicity 2 (sST2). The main function of sST2 is to sequester IL-33. As a result, IL-33 is not available to membrane bound ST2 (mST2) on Th2 cells and ST2^+^FoxP3^+^ Treg cells. Blocking of ST2 in peritransplant period attenuated GVHD severity and lethality (103).

A proper diagnosis is crucial for GVHD management. Recently, increased serum ferritin levels in allo-HCT patients have been correlated with GVHD, suggesting that ferritin can serve as a diagnosis marker in combination with other laboratory markers (104). HMGB1 is a mediator of inflammation that plays an important role in Treg/Th17 homeostasis. HMGB1 expression is reported to be positively correlated with aGVHD severity and may therefore also serve as diagnostic marker (105). In addition, several microRNAs may serve as biomarkers as GVHD. For example, the main function of miR-181a is modulation of T cell function via downregulation of IFN-γ. Interestingly, the level of miR-181a reduces significantly prior to the onset of aGVHD and its reduction seems to indicate the severity of aGVHD (106). Significant levels of another microRNA, miR-586, were observed in plasma at day 7 post allo-HCT and miR-586 could be a potential biomarker for predicting aGVHD and may also be targeted for GVHD management (107).

Due to serious side effects of the currently practiced regimens such as MMF or MTX, new therapeutic targets with less side effects are being vigorously pursued. For example, a study carried out on cyclosporine A based GVHD prophylaxis with enteric-coated mycophenolate sodium instead of MMF or MTX reported to reduce GVHD with less side effects (108). Another major problem in GVHD management is drug resistance, especially, SR. Therefore, a study carried out to test genetic disruption of the glucocorticoid receptor gene. This study provides clinical protocols for producing and administering high-purity genetically-engineered virus-specific T cells that are resistant to the suppressive effect of corticosteroids (109). In addition, Ruxolitinib, a JAK1/2 inhibitor has been found effective in SR patients with aGVHD and cGVHD (110). The αβTCR is highly important in the pathogenesis of GVHD because it is the primary signal for activating T cells. The humanized MoAb of GZ-αβTCR attenuates the function T cells, suppresses clinical signs of GVHD and increases the survival of patients (111). It is most desirable that allo-HCT may cause high GVL effect and negligible GVHD. A recent study has shown the importance of cytolytic T cells in the enhancement of GVL response. The adoptive transfer of naïve donor-derived CD8^+^ cytolytic T cells has evolved as a promising strategy to improve GVL effect (112).

In conclusion, the recent development in GVHD therapy and diagnosis has opened a new dimension of innovative strategies toward a potential cure for the adverse side effects of allo-HCT. However, further study is required to bring these novel strategies to the bedside.

## Future Perspectives

Allo-HCT has demonstrated a beneficial impact for patients suffering from various health ailments. However, prevalence of GVHD either acute or chronic poses a severe health concern and remains a major obstacle for more successful application of allo-HCT. Although a number of therapeutic options are currently available to manage GVHD, these regimens have serious adverse effects. Due to the central role of T cells in GVHD pathogenesis, most of the therapeutic regimens are targeted at TCD and T cell suppression. However, because T cells are essential in tumor immunity and infection control, these strategies of overall T cell suppression undesirably compromise host health. Therefore, more specific modulation of T cell function is required for successful GVHD treatment. In this setting, further elucidation of how co-stimulatory and co-inhibitory molecules modulate allogeneic immune response may reveal feasible targets that can bring beneficial outcomes. Of note, recent mouse models and human studies have demonstrated that intestinal microbiota are involved in inducing GVHD and one potential mechanism is influencing reconstitution of various T cell subsets after allo-HCT (113–115). It remains to be determined whether microbiota may modulate T cell co-signaling during allo-HCT.

Past studies have clearly demonstrated the important roles of several co-stimulatory/co-inhibitory molecules in GVGD, including CD28, CTLA-4, PD-1, OX40, and CD27. However, many other molecules are yet to be studied for their impact in allo-HCT. For example, leukocyte-associated Ig-like receptor (LAIR) molecules belong to the Ig-SF that contain one Ig-like domain and two cytoplasmic ITIM domains. These LAIR molecules may function as an inhibitory receptor on NK cells, T cells, B cells, monocytes, DCs and most thymocytes (116). CD96, also known as Tactile (T cell activation, increased late expression) is expressed on CD4^+^ and CD8^+^ T cells, NK cells and also present on selected B cells (117). Human CD96 interacts with nectin and nectin-like proteins and regulates NK cell function (118). CD160 is found on a subpopulation of cytolytic T cells and NK cells and functions as a broad specificity receptor for MHC I and associated molecules. The binding of CD160 to MHC I resulted in inhibition of cell-mediated cytotoxicity by CD8^+^ T cells and NK cells (119). Considering the roles of T cells and NK cells in GVHD, these molecules could be involved in the pathogenesis of GVHD. CD200, also known as OX-2, is another Ig-SF membrane glycoprotein primarily expressed on myeloid lineage and inhibits myeloid cell activity (120). The involvement of myeloid cells in allo-HCT suggests that CD200 may play a role in GVHD. The CD300 family of molecules, also known as IREM (immune receptor expressed by myeloid cells), possess paired activating and inhibitory receptor functions and recognize lipids exposed on the outer leaflet of plasma membrane of dead and activated cells (121). Their ability to tune leukocyte function and immune responses suggests potential involvement in GVHD. The butyrophilin (BTN) and BTN like (BTNL) co-stimulatory family members are structurally similar to B7 family but are functionally different (122). BTN or BTNL family members are involved in immune regulation but their role in GVHD is yet to be explored. The signaling lymphocyte activation molecule (SLAM or CD150) family is a subset of the CD2 family of receptors that can either promote or inhibit the function of primary activating receptors (123). How SLAM is involved in GVHD is unknown. Taken these examples together, the roles and mechanisms by which many of these less studied co-signaling molecules regulate GVHD are largely undefined. Many concerted studies are needed to determine whether these molecules can serve as potential therapeutic options for successful treatment of GVHD.

## Concluding Remark

**Figure 1:**
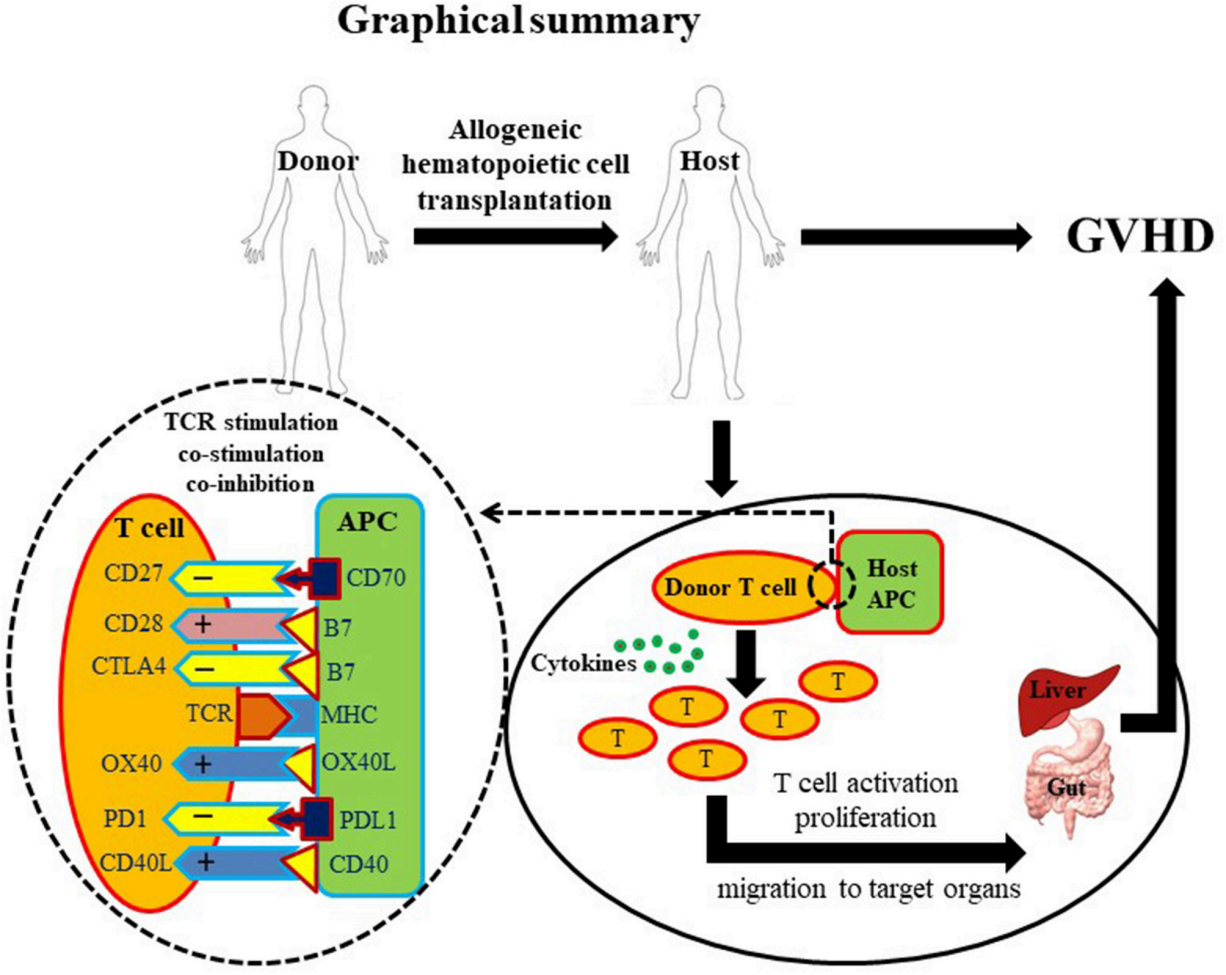
Graphical summary. A diagrammatic illustration of GVHD occurrence post allo-HCT. Briefly, a healthy donor provides hematopoietic cells to a diseased host. Post-transplantation, donor-derived T cells activate, proliferate and migrate to target tissues. T cell activation requires three basic signals: first, T cell receptor/MHC signal; second, co-stimulatory signal; and third, cytokines. Due to the critical role of T cells in GVHD pathogenesis, T cell depletion (TCD) and suppression account for most established therapeutic approaches. However, overall T cell depletion and suppression may give rise to a disease prone host. Co-stimulatory molecules (depicted with + signs) are highly important for optimal activation of T cells. In contrast, co-inhibitory molecules (depicted with – signs) are important to down-regulate T cell activation preventing excessive immune response. Innovative manipulation of co-stimulatory/co-inhibitory signals may represent a more specific approach to control GVHD.

In summary, the potential benefits of allo-HCT are offset by the incidence of GVHD. The current therapeutics based on TCD or T cell suppression are partially effective to control GVHD but carry serious side effects. Co-stimulatory and co-inhibitory pathways involved in T cell function have shown substantial significance in GVHD pathogenesis (Figure 1). Further intensive and extensive exploration of these pathways is needed before these potential therapeutic targets become new clinical options to cure GVHD without causing severe side effects.

## Author Contributions

All authors listed have made a substantial, direct and intellectual contribution to the work, and approved it for publication.

### Conflict of Interest Statement

The authors declare that the research was conducted in the absence of any commercial or financial relationships that could be construed as a potential conflict of interest.

## Abbreviations

Allo-HCT: allogeneic hematopoietic cell transplantation
BMT: bone marrow transplantation
IL: interleukin
TNF-α: tumor necrosis factor alpha
IFN-γ: interferon gamma
TGF: transforming growth factor
GVHD: graft-vs.-host disease
GzmB: granzyme B
MLN: mesenteric lymph node
TCD: T cell depletion
MHC: major histocompatibility complex
TCR: T cell receptor
PD-1: programmed cell death protein 1
CTLA-4 or CD152: cytotoxic T-lymphocyte-associated protein 4
MoAbs: monoclonal antibodies
DC: dendritic cells
APCs: antigen presenting cells
CD: cluster of differentiation
Treg: regulatory T cell
iNKT: invariant NKT
ITAMs: immunoreceptor tyrosine-based activation motifs.
